# Supplementary figures and images for: Regulating synchronous oscillations of cerebellar granule cells by different types of inhibition
Source: PLoS Comput Biol. 2021 Jun 28;17(6):e1009163. doi: 10.1371/journal.pcbi.1009163 (PMC8270418; doi:10.1371/journal.pcbi.1009163)

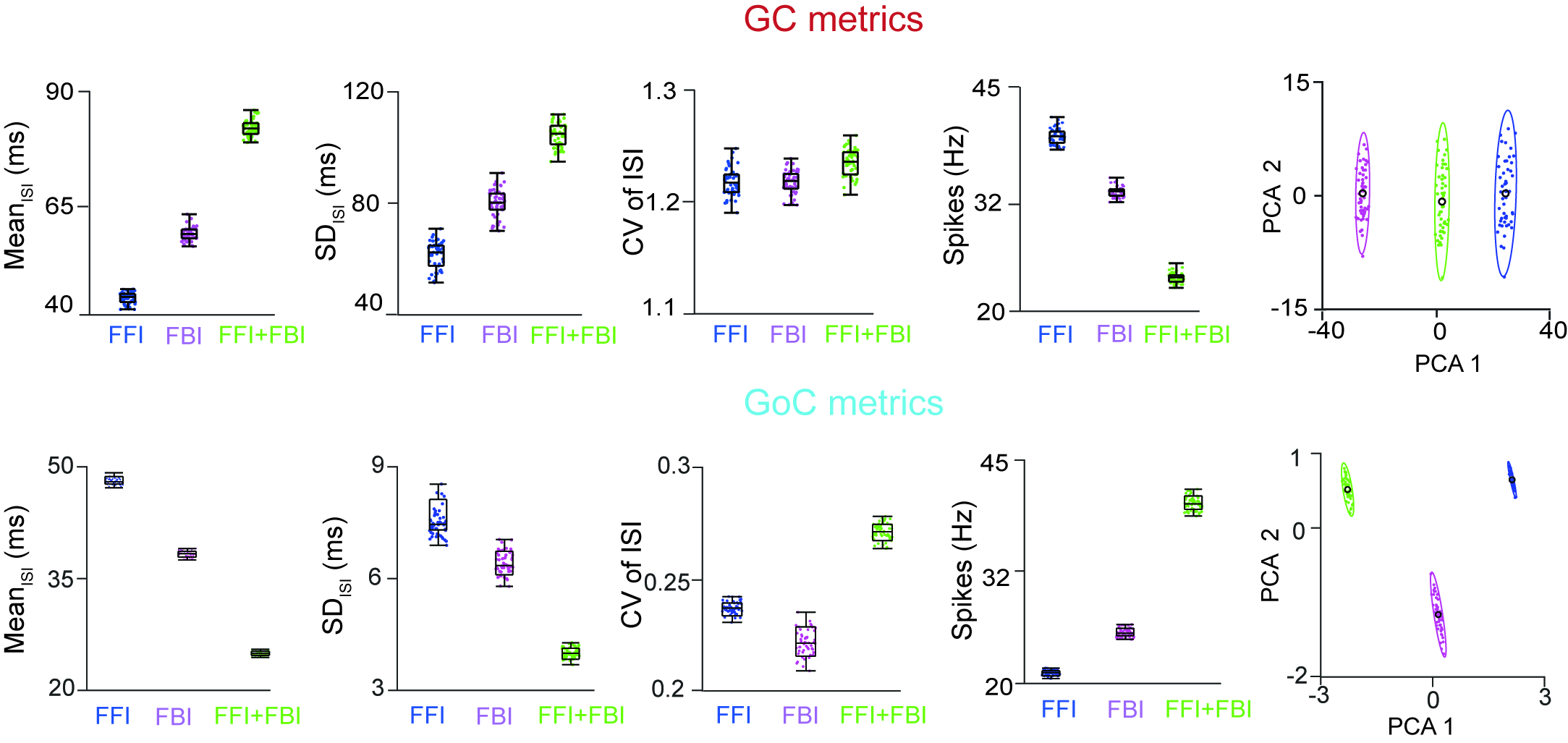

Supplement: S1 Fig — Diverse firing patterns of GCs (top) and GoCs (bottom) induced by feedforward and feedback GoC inhibition. (Left four panels) Characteristics of spike responses in three scenarios. Box plots represent quartiles (minimum, 25%, median, 75% and maximum values) for the mean of inter-spike intervals (ISI), standard deviation (SD), coefficient of variation (CV), and average spike rate, of 50 trials. Each trail (each data point) is a 10 second population firing rate averaged over all GCs or GoCs. The stimulus to the network is 50 Hz Poisson input. (Right panel) Neural responses are distinct across three scenarios visualized by principal component analysis (PCA) of population spikes of 50 trials. Black circles are the cluster centers and colored ellipse outlines indicate 95% confidence intervals calculated by the k-means clustering algorithm. (TIF) [file pcbi.1009163.s001.tif]

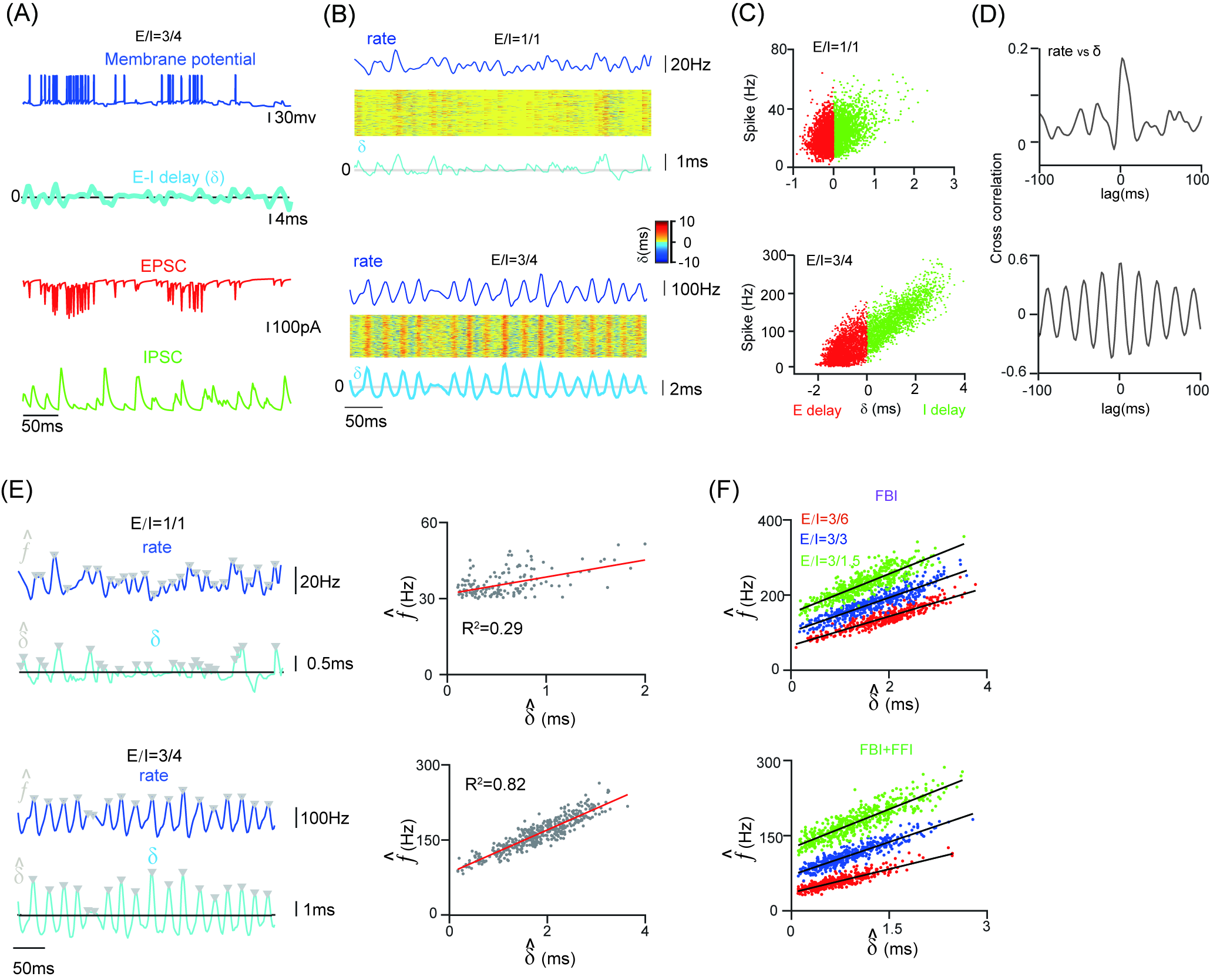

Supplement: S2 Fig — The relationship of the GC firing rate and excitation (E) and inhibition (I). (A) Time courses of the membrane potential, MF-evoked EPSC (Vhold = −70 mv), GoC-evoked IPSC (Vhold = 0 mv) and E-I delay obtained as cross-correlations between EPSC and IPSC of one GC. The Poisson stimulus is at 25 Hz. Idelay > 0 means the I delay, otherwise Idelay < 0 as the E delay. (B) Time courses of GC firing rate, raster plots of δ of all GCs and the averaged δ over all GCs in two conditions of E/I = 1/1 (without oscillation) and E/I = 3/4 (with oscillation). (C) The GC population firing rate as a function of E-I delay averaged over all GCs, i.e., the average of the rater plot in B. (D) Cross-correlations of firing rate and δ. (E) The peaks of firing rate (f^) and amplitudes of oscillation (δ^). (F) f^ as a function of δ^ in different E-I combinations with FBI and FEI+FFI. Linear fits indicated by red solid lines. (TIF) [file pcbi.1009163.s002.tif]

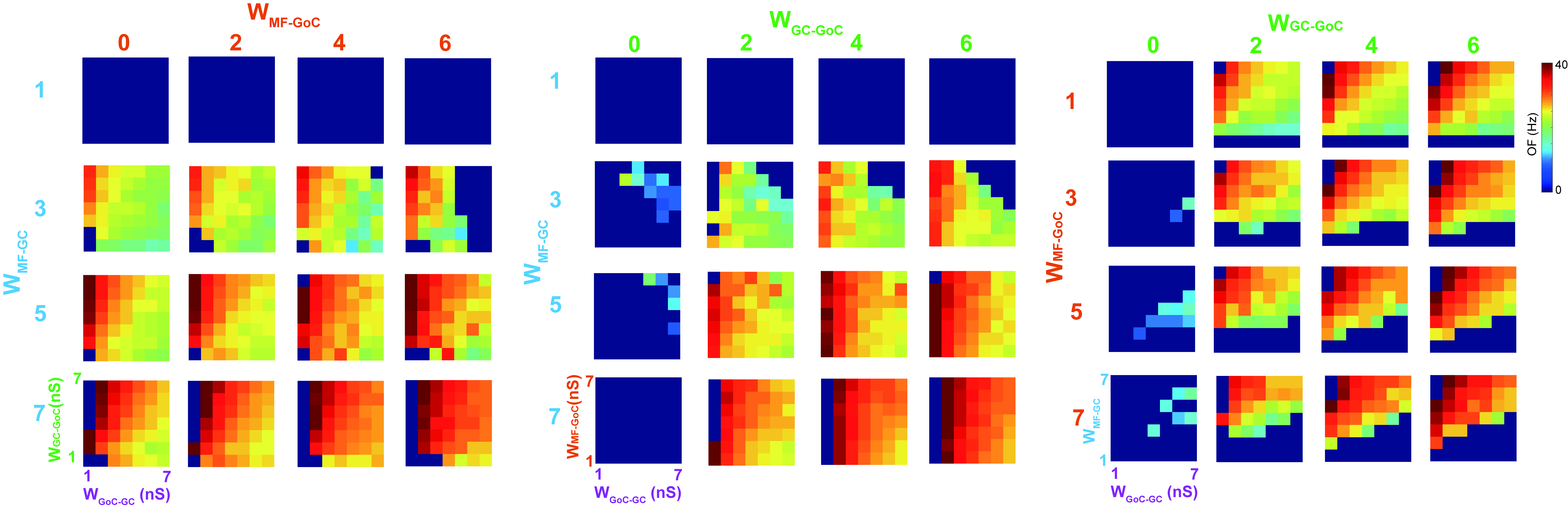

Supplement: S3 Fig — The change of oscillation frequency (OF) under a range of weights in different synapses. (TIF) [file pcbi.1009163.s003.tif]

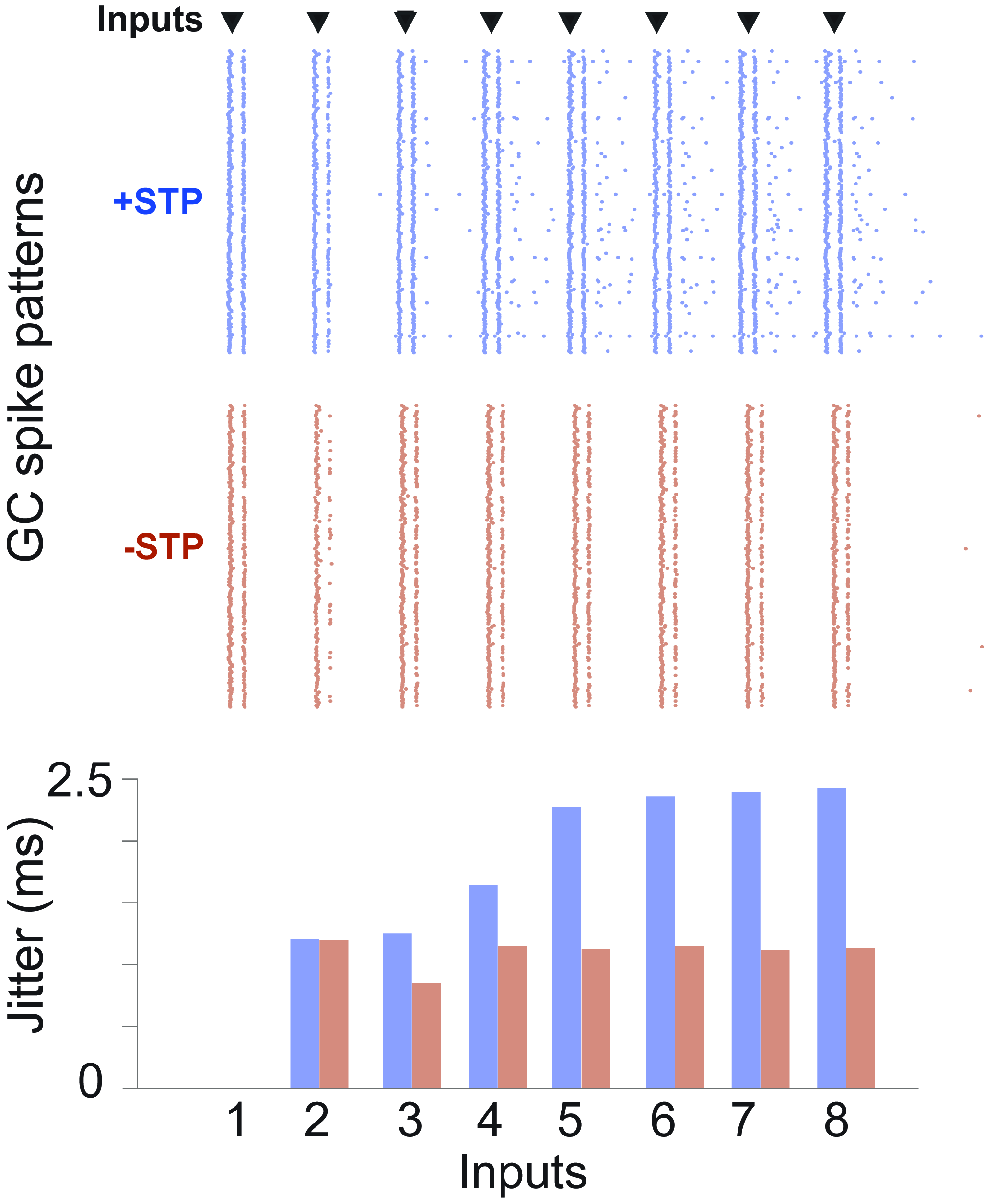

Supplement: S4 Fig — Spike time jitters of GCs changed by the GoC-GC synapses with STP (blue) or without STP (red). (TIF) [file pcbi.1009163.s004.tif]

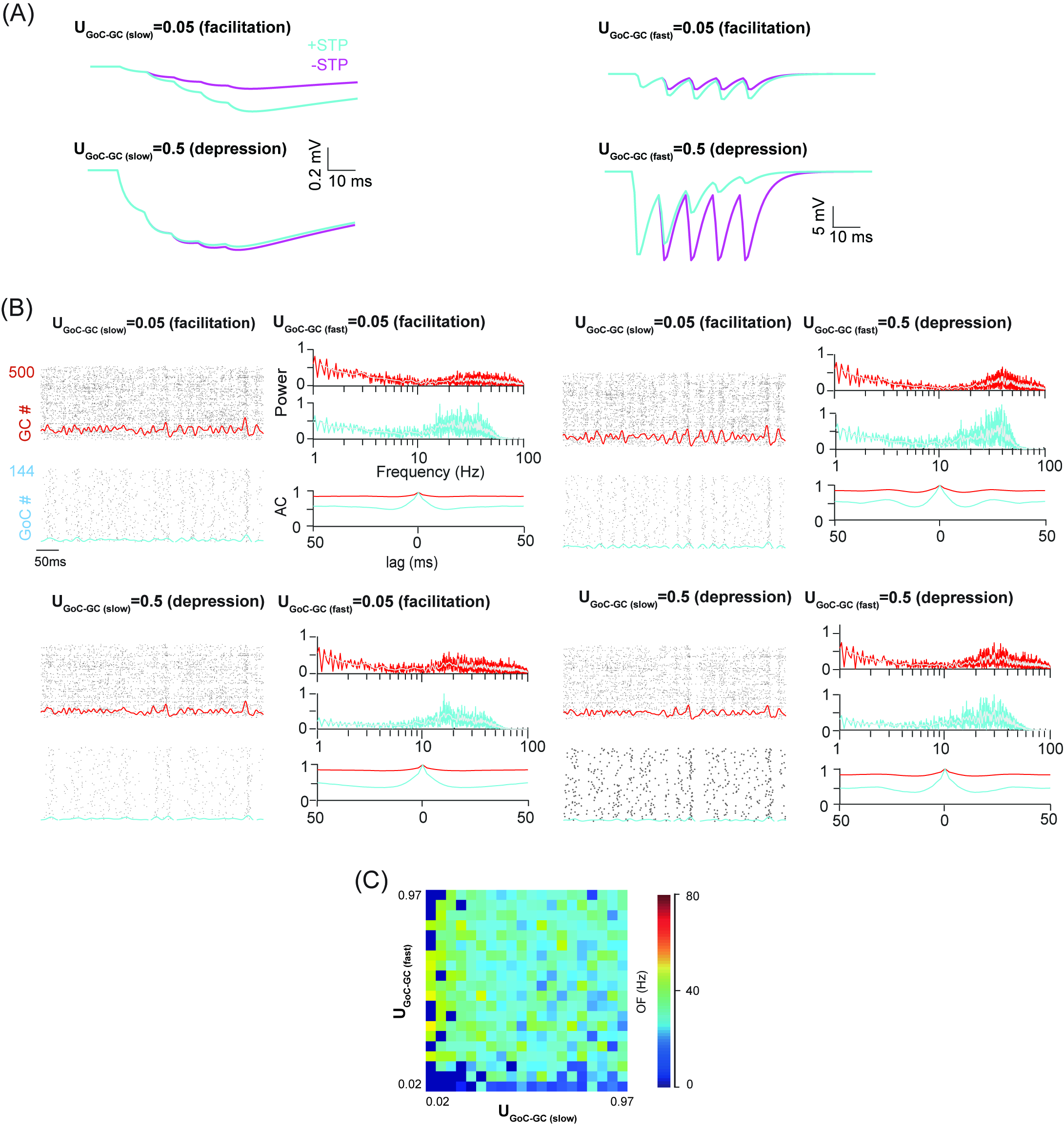

Supplement: S5 Fig — Network oscillation suppressed by short-term plasticity (STP) of GoC-GC synapses. (A) Profiles of STP facilitation and depression controlled by the STP parameter U. Inhibitory postsynaptic potentials recorded at GCs induced by GoCs for both slow and fast synaptic components. (B) Suppression of network oscillation independent on the detailed profiles of STP, either facilitation or depression, for both components of fast and slow dynamics of GoC-GC synapses. Spike raster and population firing rate of all GCs and GoCs, and the corresponding power spectrum and auto-correlation (AC) of population firing rate. (C) Oscillation frequency (OF) suppressed by STP over a range of the parameter U in GoC-GC synapses. Networks with FBI. (TIF) [file pcbi.1009163.s005.tif]

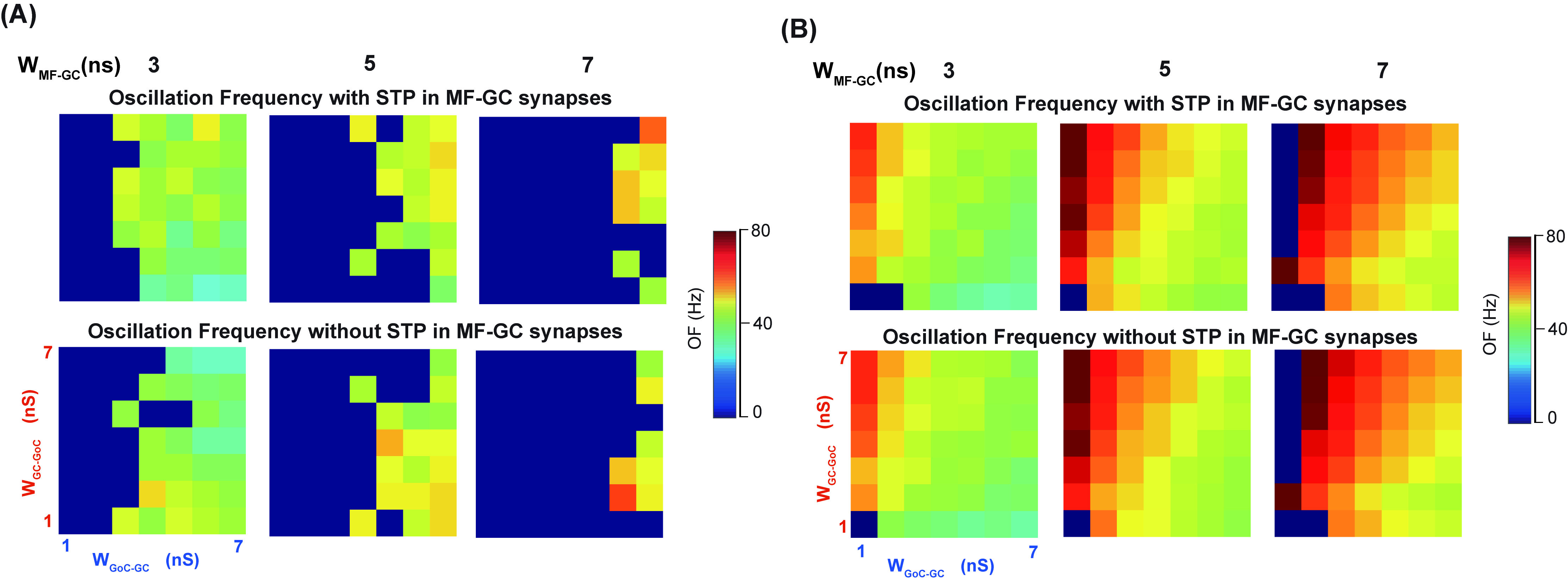

Supplement: S6 Fig — (A) Modulation of the oscillation frequency (OF) over WGC−GoC and WGoC−GC strengths at different weights of WMF−GC, under the conditions of presence and absence of the STP in MF-GC synapses. Here is the FBI network with Poisson inputs at 25Hz. The GoC-GC synaptic STP was included. (B) Similar to A, but the GoC-GC synaptic STP was turned off. The MF-GC STP has little effect on network oscillation, compared to the GoC-GC STP. (TIF) [file pcbi.1009163.s006.tif]

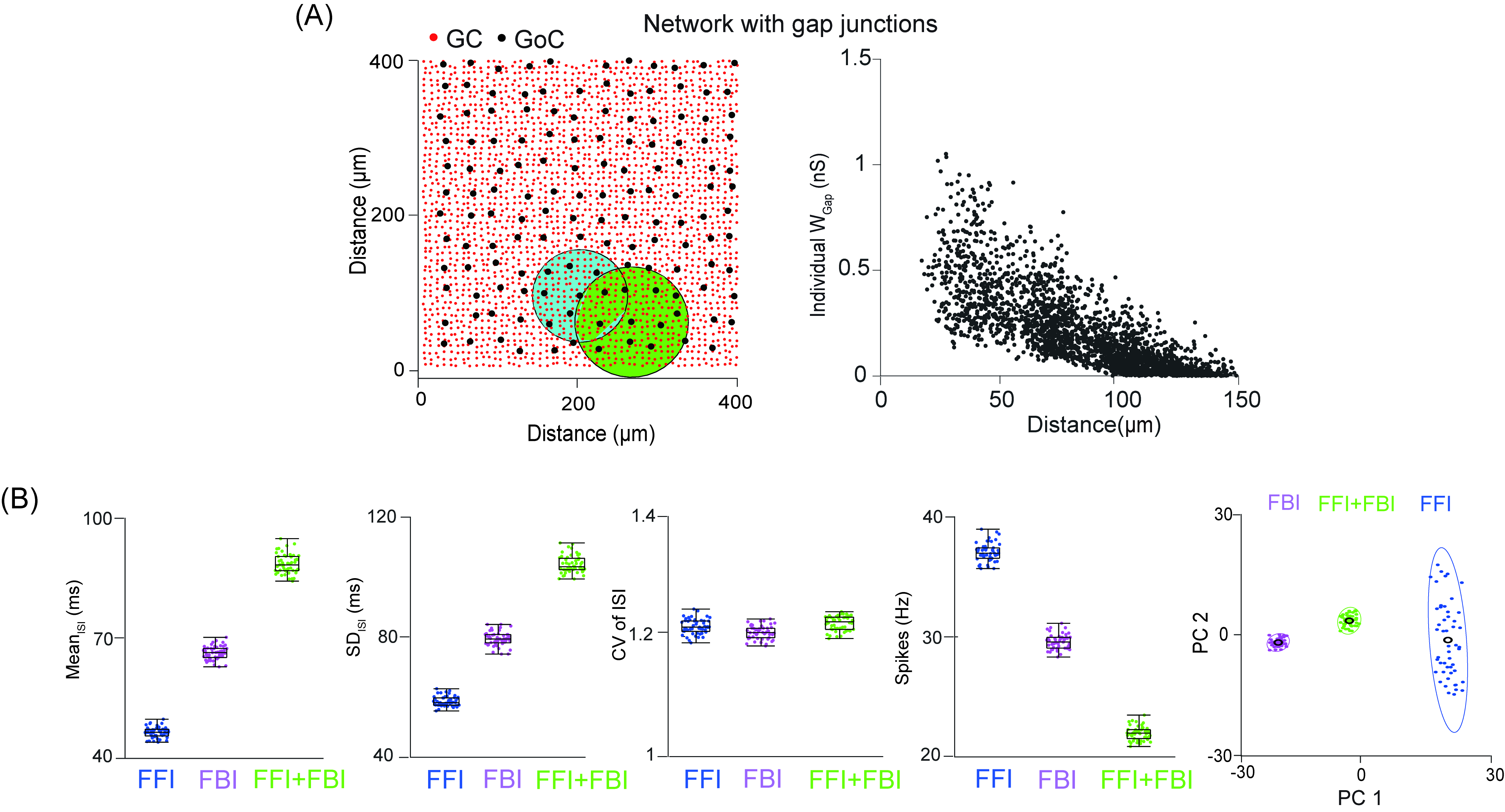

Supplement: S7 Fig — GC responses with gap junction of GoCs in the network model. (A) (Left) The layout of network model with gap junctions. A 2D grid showing the position of 144 GoCs (black dots) and 2000 GCs (red) in the model. 144 GoCs are arranged on a 12x12 grid with 33 μm spacing, where the location of each GoC was drawn randomly from the grid vertices, using a uniform distribution between ± 25% of grid spacing both in x and y directions. The radius of each GoC was drawn randomly from 0.7 to 1.3 times the average radius (70 μm) as well as its density of processes (from 0.7 to 1.3 relative to average). The extensions of two GoCs are represented by a green and cyan disk, respectively. The weight conductance between two cells was taken proportional to the area of overlap between the two disks and to the relative densities of processes of both cells. (Right) The distribution of the weights of all gap junctions as a function of the distance between GoCs. (B) Summary plots of metrics for a group of 50 trials in three network connections. Box plots represent quartiles (minimum, 25%, median, 75% and maximum values) for mean ISI, standard deviation (SD), coefficient of variation (CV), spike number. Three clusters using two principal components (PAC) and the k-means clustering methods. Black circles are the cluster centers and ellipse perimeters indicate 95% confidence intervals. All colors correspond to three scenarios of GoC inhibition. (TIF) [file pcbi.1009163.s007.tif]

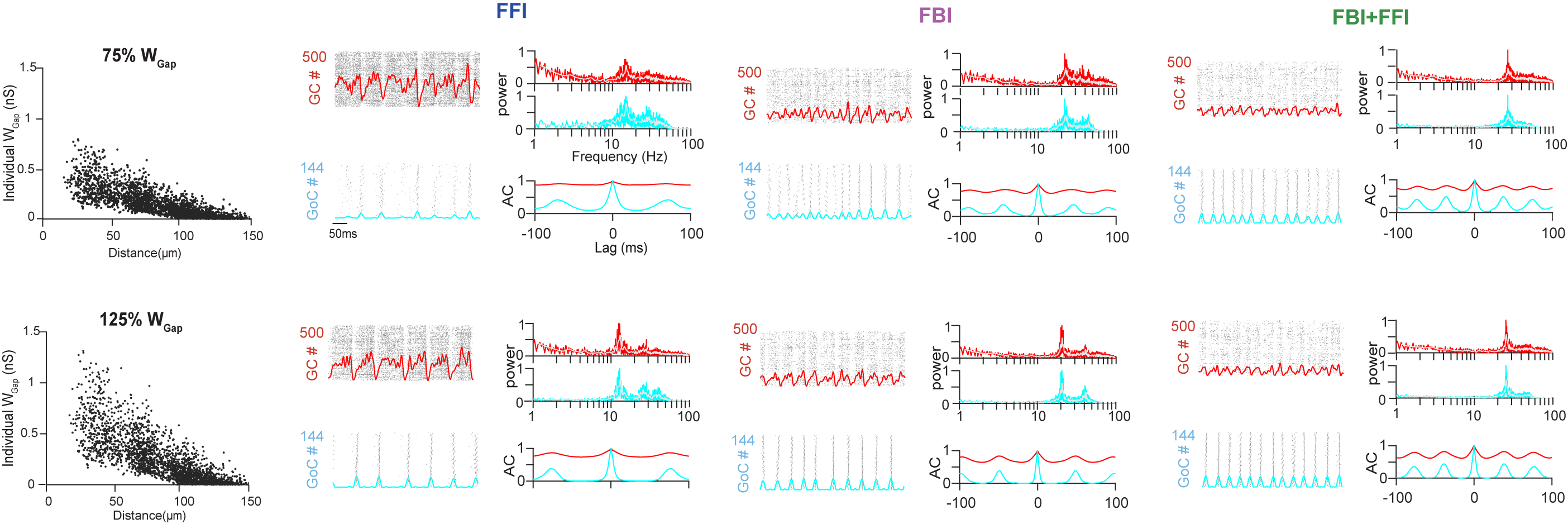

Supplement: S8 Fig — Network oscillation boosted by gap junction at different levels of gap junction strengths (top, reduced weight as 75% compared to the default case shown in S7 Fig; bottom, increased weight as 125%). Spike raster and population firing rate of GCs and GoCs, and the corresponding power spectrum and auto-correlation (AC) of population firing rate in three network scenarios of FFI, FBI, and FBI+FFI. GoC-GC STP included. (TIF) [file pcbi.1009163.s008.tif]

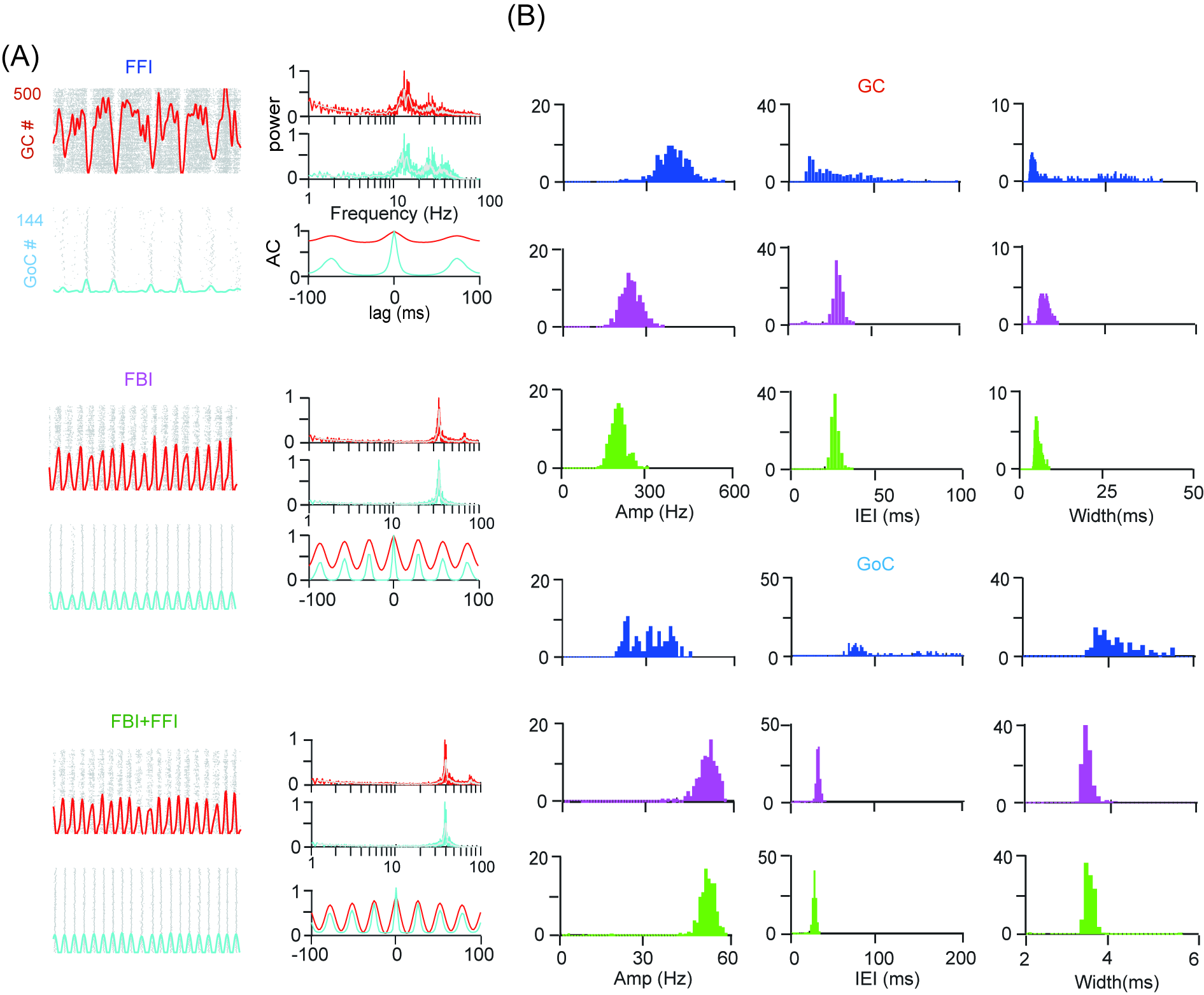

Supplement: S9 Fig — Network oscillation with gap junctions in GoCs, but without GoC-CC synaptic STP. (A) The spike raster and population firing rate of GCs and GoCs (left), and the corresponding power spectrum and autocorrelation (AC) of population firing rate (right), in three network scenarios of feedforward, feedback and both types of inhibition. (B) Network oscillation metrics characterized by the amplitude (Amp), inter-event interval (IEI) and width. Histograms of three metrics for GC (top) and GoC (bottom). (TIF) [file pcbi.1009163.s009.tif]

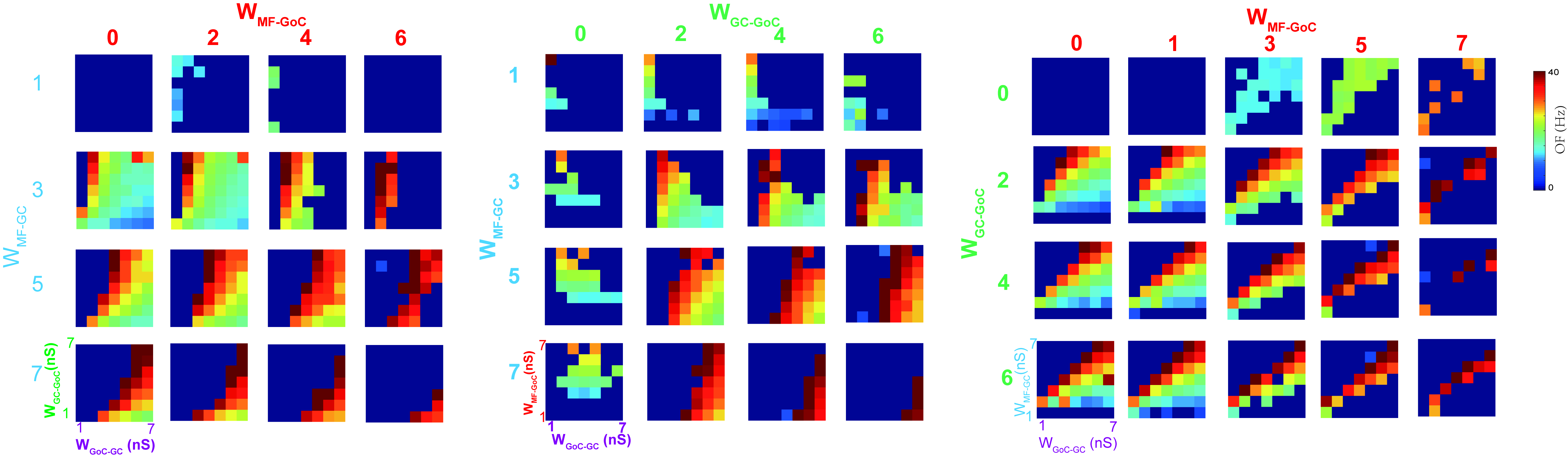

Supplement: S10 Fig — The change of oscillation frequency (OF) with different synapses, under the condition of gap junction included. (TIF) [file pcbi.1009163.s010.tif]

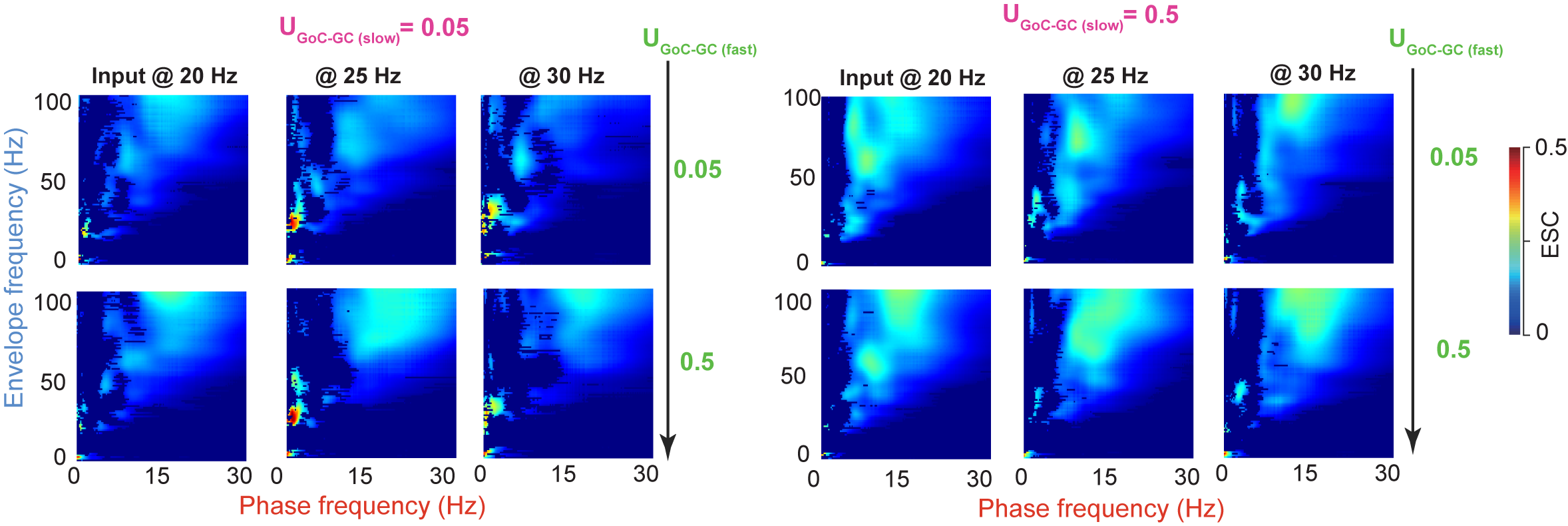

Supplement: S11 Fig — Cross-frequency coupling of network oscillations impaired by STP when gap junctions are not included. The ESC measure as a function of frequencies of phase (low-pass filter) and envelope (high-pass filter) computed for GC oscillations with Poisson inputs at 20, 25, and 30 Hz under the scenario of FBI. Different profiles, facilitation or depression controlled by the parameter U, of STP have similar effects on ESC. (TIF) [file pcbi.1009163.s011.tif]

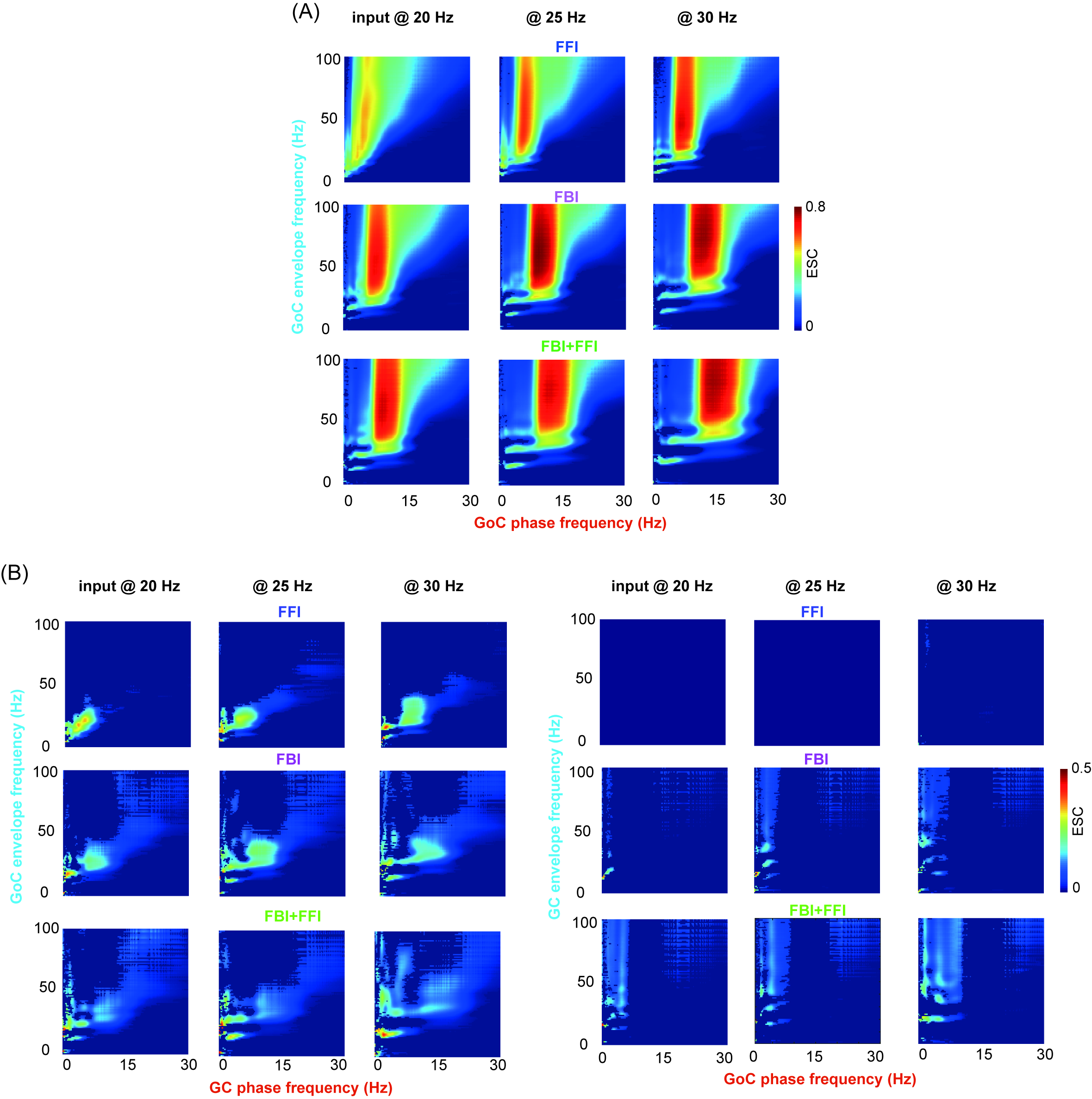

Supplement: S12 Fig — The Cross-frequency coupling in the population of GoCs and between GoCs and GCs. (A) The strength of phase-amplitude coupling (PAC) quantified for GoCs oscillations with different Poisson inputs at 20, 25, and 30 Hz under three types of network connections. (B) Same as in (A), but for PAC between GCs and GoCs. (Left) PAC between GCs (phase) and GoCs (envelope). (Right) PAC between GoCs (phase) and GCs (envelope). (TIF) [file pcbi.1009163.s012.tif]
