# Supplementary material for: Regulating synchronous oscillations of cerebellar granule cells by different types of inhibition
Source: PLoS Comput Biol. 2021 Jun 28;17(6):e1009163. doi: 10.1371/journal.pcbi.1009163 (PMC8270418; doi:10.1371/journal.pcbi.1009163)
Supplement: Attachment — Submitted filename: PCB_rebuttel.pdf [file pcbi.1009163.s014.pdf]

PLOS Computational Biology (PCOMPBIOL-D-21-00018)

Dear Editor and Reviewers:

First of all, we would like to express our gratitude to you for carefully reading our manuscript and giving us constructive suggestions. We have thoroughly considered your comments, and revised our manuscript in light of your remarks and suggestions. Thank you all for your comments during the revision of our manuscript.

The following are point-to-point responses to the reviewers' comments. The reply is arranged in Q's and A's. We first quote your comments, and then give our answers after each of your questions.

Replies to the Comments from Editors:

From reading the reports, all the points raised seem reasonable and should be addressed in a reworked manuscript. In particular, the reviewers were concerned about clarity of the main message, as well as model choices. A revised manuscript should have substantial effort devoted to robustness tests and/or motivation of the parameters.

We have carefully revised our manuscript in light of the comments provided by the reviewers. We revised our manuscript significantly to improve the clarity of the main message and model choices, added more analysis with additional parameter settings for robustness tests (Supplemental Fig.S5, Fig.S8, and Fig.S11), and extended our Discussion with two new parts (subsections D and E).
We provided a version of the revised manuscript with highlights denoting where the text has been revised (in red) for easy reviewing. Please find the marked PDF file uploaded as '**Revised Article with Changes Highlighted.pdf**'. All the responses below indicated by line numbers refer to the marked PDF file rather than the clean copy PDF file.

Thank you very much.

Reviewer #1:

Inhibitory cells and gap junctions play a crucial role for many forms of neural oscillations – that is long known – but the detailed synaptic mechanisms are often not clear. The authors present a network model to test several excitatory/inhibitory conditions between mossy fibres, Golgi and granular cells in the cerebellum. They showed that feedback inhibition between Golgi and Granular cell is important to establish network oscillations, whereas gap junctions between Golgi cells maintain

robust oscillations. Overall, the results are sound and the findings interesting, but the manuscript needs to address some urgent points, especially the novelty point, to proceed.

Q-I-1: The main experimental data and major parts for the computational cell and network model are based on Dugue et al, 2009. The same paper also states the importance of gap junction coupled Golgi cells for oscillation. The here submitted manuscript needs to clarify where the novelty is compared to the Dugue.

A-I-1: Here our work is more focusing on Granular cells (GCs) and their interaction with Golgi cells (GoCs). The work of Dugue et al, 2009 (D09) was focusing on the detailed GoC gap junctions measured experimentally and set up a network model of GoCs without modeled GCs. Our work here is to use their evidence of GoCs and the knowledge of gap junctions, examine the interaction between GCs and GoCs in detail, and show how different formats of GoC inhibition change the network dynamics of GCs. We revised the text to make this point clear.

Q-I-2: The authors do not provide experimental work alongside with the theoretical work to validate the results. Instead, they used already published experimental data to setup the model framework (see above). In my opinion it would be good to provide more evidence the findings by comparing to other models or systems. For example, the oscillations in the retina are well studied.

A-I-2: Thanks for the suggestion. Indeed, our work here is a theoretical study as we are unable to conduct the corresponding experiments. However, as the reviewer noted, the phenomena of oscillations and systems of excitatory cells (GCs) and inhibitory cells (GoCs) in our work have a general implication to other models and systems. We now have discussed the implication of our findings to other systems, and added a new part in Discussion for this point (see line 349 subsection D - ‘Implications for other systems’).

Q-I-3: I was astonished that STP does not have a stronger effect on the oscillations. Spike-Timing Dependent Plasticity was shown in several papers to have a profound effect on oscillations at least in a feed forward model (Luz and Shamir 2016, PloS Comp Biol). I feel you should extend the discussion on that topic.

A-I-3: Thanks for the suggestion. Short-term plasticity (STP) used here indeed has a stronger effect on the oscillations. To examine this point in detail, we systematically investigate the effect of STP by manipulating its parameters and changing the profiles of facilitation and depression, and we added these new results in Fig. S5 and Fig.S11.
Thanks for the reference, we have included it and extended our discussion with a new part in the Discussion Section (see line 349 subsection D - ‘Implications for other systems’).

Q-I-4: Can your model inflict seizure like oscillations? Or better asked: which parameter/condition needs to be changed to go ictal? That would be interesting for a wider audience.

A-I-4: Thanks for the question. At this stage, our model was not designed for addressing ictal seizures. As for the seizure-like oscillations, it will need additional and specific mechanisms for network modeling. We are happy to report our future studies on this topic through a detailed study on the interaction of the cerebellum with other brain areas.

Reviewer #2:

The authors present modeling results addressing several interrelated questions concerning oscillations and synchrony in the cerebellum: how do feedback and feedforward Golgi cell inhibition differentially contribute to synchrony? How do gap junctions and chemical synaptic dynamics affect synchrony? And does the resulting oscillatory network state exhibit cross-frequency coupling?

While these are all interesting and relevant questions, and the modeling work shows potential, it fails to provide broader insights or conclusions regarding these phenomena. The conclusion "the interaction of various types of inhibition plays a crucial role in regulating synchronous oscillations of neural populations" is vague and likely true for essentially any system. While there are some more specific conclusions, they are essentially of the form "this phenomena was observed in this specific parameter range," which fails to illuminate either mechanisms or functional relevance. These conclusions could be expanded into broader, impactful insights by several paths:

Q-II-1: Looking for explanations underlying the network-level trends: Can the effects of varying different coupling strengths be understood with a simple linear interaction model? How exactly does the STP desynchronize? What is the mechanism for cross-frequency coupling?

A-II-1: Thanks for the questions. As here we are examining detailed synaptic mechanisms, a simple linear interaction model using firing rate neurons is not suitable for this purpose. To future address the questions of STP and cross-frequency coupling, we conducted additional analysis by systematically varying the parameters of the STP, added new results in Fig. S5 and Fig.S11, and extended our discussion (see line 349 subsection D - 'Implications for other systems', and line 372 subsection E – 'Limitations').

Q-II-2:Expanding on the functional relevance of the network synchrony by, for instance, linking the model to adaptation of Purkinje cell output. (See comment below on reconciling the synchronized vs diverse-timescale views of granule layer function)

A-II-2: Thanks for the comment. Indeed, the network synchrony of the granular layer has an important role in affecting the dynamics of Purkinje cell output. We had a recent work related to this point.

Tang Y., An L., Yuan Y., Pei Q, Wang Q., Liu, J. K., Modulation of the dynamics of cerebellar Purkinje cells through the interaction of excitatory and inhibitory feedforward pathways, PLoS Comput Biol 17(2): e1008670 (2021)

It is from the above work, we found the synchrony of GCs has a strong effect on the dynamics of Purkinje cells. There we explored the consequence of the synchronized granular layer input on Purkinje cell output. However, there was no detailed mechanism of the granular layer network examined in that study.

In this work, we went a step further, and conducted a detailed study on the phenomena of the synchrony of GCs, together with the effect of GoCs. As the reviewer noted, the functional relevance of the network synchrony could be further explored, not only considering downstream Purkinje cells, but also the interaction with other parts of the brain where the output of Purkinje cells plays a role. We are working on extending our model to include a more functional model of the cerebellum with the granular layer network and Purkinje cell network. We are also working on the cortico-cerebellar interaction. All these ongoing work have the potential for comparing with experimental results to examine the capability of network synchrony. For the current revised manuscript, we extended our discussion with a new part in the Discussion Section (see line 349 subsection D - 'Implications for other systems').

Q-II-3: Connect the modeling work more directly to the past modeling work it builds on by more precisely identifying what questions were left open and how this work resolves those questions. This may have been intended, but wasn't clear to me. This might require more precisely matching aspects of the model to the relevant previous work.

A-II-2: Thanks for the comment and sorry for the confusion. Indeed, our work is using and based on the past work on network modeling of GoCs (see *Q/A-1-1* from Reviewer 1). To better explain this point, we added a part in the Discussion Section with a detailed discussion on the relevance of our work with previous work, in the context of both cerebellum and general neural network modeling of oscillations (see line 349 subsection D - 'Implications for other systems').

I also briefly note several specific issues found in the manuscript that are less comprehensive, but still critical to address.

Q-II-4: L78: "excitation and inhibition have contrary effects on the oscillation frequency, which rises with increasing excitability, and decreases as increasing inhibition" – this in particular is a conclusion that seems possibly explainable by considering the oscillation frequency of a simple linear neural mass model of the two coupled populations.

A-II-4: We agree with the Reviewer regarding this point. A number of previous and classic theoretical and modeling works have been developed to address the question of oscillation by using two coupled populations of firing rate units of excitation and inhibition. Here our focus is on the role of detailed synaptic dynamics, including nonlinear short-term plasticity and gap junction, in regulating network dynamics. Thus, we employed the current approach with spiking neurons. The aim and advantage of this approach is to explain experimental observations with detailed mechanisms that then can be tested thereafter by our experimental collaborators.

Q-II-5:L92: "[UBCs] operate like intrinsic MFs within the granular layer to diversify inputs and can be represented as phase shifted MFs [36]," – One of the primary references for previous modeling that this builds on is on GC-UBC interactions, in which the presence of new timescales/phase shifts of activity in the UBCs, not found in MF input, is shown to be important. Yet the authors claim modeling all MF and UBC input as Poisson spiketrains with the same properties is sufficient. This decision should be better justified, or they should be included in the model somehow.

A-II-5: Thanks for the comment and sorry for the confusion. Indeed, UBCs as a unique cell type has been previously studied experimentally and theoretically by us and other groups. The best scenario for studying UBCs is the vestibular system, as UBCs are mostly distributed in the area of the cerebellum related to it. UBCs are the basis for diverse time-scale dynamics of the vestibular system. Taken with other points revealed by the Reviewer (*Q/A-II-7*), we now moved this part of the description of UBCs to the Discussion Section, and added a new part in the Discussion Section to extend our discussions (see line 372 subsection E - 'Limitations').

Q-II-6:L112: the reason for analyzing the network data by k-means clustering is unclear. While this does emphasize the differences in activity between the three forms of inhibition, clustering is more appropriate if the identity of the groups are not known in advance. The same view of the activity space could be used without clustering, as the distinctions are quite clear by eye (although what form of data exactly PCA is applied to should be clarified).

A-II-6: Sorry for the confusion. We fully agree with the Reviewer. As noted by the Reviewer, the distinctions are clear by eye. We used K-means clustering only for visualization, as it enables us to integrate all the characteristics (those measures of mean_ISI, SI_ISI, CV of ISI, and spike number) of firing activities. Thus, we moved this part to Supplemental Fig. 1, and integrated the remaining of Fig.1 into Fig.2 as a new Fig.1 in the revised version. In this way, the text is more concise.

Q-II-7:L122 "potential function for enhancing sensory representation and facilitating pattern separation 122 for downstream cells in the cerebellum." - In distinct areas of work on the cerebellum, researchers may view the ideal functional state of the granular layer network as synchronized to enable effective motor control (e.g. the work on Golgi gap junctions this builds on) or as containing a diverse range of timescales/patterns to enable learning of precise timing (e.g. the work on UBCs this builds on). This work seems to only consider the former, but since it builds on both views and describes the conclusions as relevant for the function of the network as a whole, they should at least discuss this contradiction. Would a real network operate with multiple subnetworks that are each synchronized, or by generating a range of patterns on top of a globally synchronized 'clock' frequency?

A-II-7: Thanks for the comments. We agree that this is an important question. It has been shown that the granular layer could be an ideal synchronized state or a state with a diverse range of timescales. We now added two new parts in the Discussion Section to extend our discussion (see line 349 subsection D - 'Implications for other systems'; line 372 subsection E - 'Limitations').
Briefly, in recent years, the studies on the cerebellum have recognized that there are multiple functional roles of the cerebellum for many types of behaviors, from traditional motor control to higher cogitative behaviors. There are roughly nine lobules organized on the whole cerebellum. It has been suggested that different functions of the granular layer are also in line with different lobules. As note by the Reviewer here, one particular and well-studied example of multiple time-scale dynamics is the unipolar brush cell, a relatively new cell type targeting to GCs. These cells, largely distributed in the lobules for the vestibular system, are suggested as internal mossy fibers or relay cells to diversify the time scale of external vestibular signals and form both excitatory and inhibitory dynamics over fast and slow time scales for the Purkinje cell output. Thus, it could be reasonable that there are multiple subnetworks in the cerebellum, and each of which plays a different function so that some are more synchronized and some more diverse, depending on the lobules and information to be processed. We extended our discussion with relevant references. The comment made by the Reviewer could be further explored in detail to take into account these aspects with considerable new efforts. We are happy to investigate this question, presumably with a collection of different experimental observations.

Q-II-8:The authors in many places refer to gap junctions as providing inhibition or "a balance of excitation and inhibition" – that is maybe technically true, but neglects the simpler intuitive explanation that gap junctions typically synchronize by providing diffusive coupling (the effects act to cancel any difference in voltage)

A-II-8: Sorry for the confusion. Indeed, gap junctions are entirely different, in terms of the balance of excitation (E) and inhibition (I). When we are talking about the E-I balance here in our work, we are simply referring to the driving input from the viewpoint of GCs, e.g., GCs receive excitatory inputs from mossy fibers and inhibition inputs from GoCs. These two streams of input are described by the weights of each type of synapse. We have carefully revised the text about this point to avoid confusion.

Q-II-9:The authors consistently use the term 'oscillations' to refer only to population-level synchronized oscillations (as would be experimentally observable via LFP etc). It would add clarity to distinguish in which scenarios or parameter regimes there may be individual elements in the network with oscillatory activity that is not synchronized. See L68: "gap junctions between GoCs can also generate oscillations [26], [32], [34], [35]." – in some of these references, gap junctions are not exactly generating oscillations, but rather synchronizing single-cell oscillations to enable network oscillation.

A-II-9: Thanks for the note. Indeed, our citations are not accurate as noted by the Reviewer. Ref [32, 34] did not involve gap junctions in their studies, and Refs [26] showed gap junctions promote network oscillation. Here we intended to cite those studies in which gap junction could generate network oscillation beyond the level of single cells. The Reviewer is right that there are also intrinsic cellular mechanisms at the single-cell level to generate oscillation without coupling between cells. We revised our text and citations/references to correct this point.

Q-II-10:In the discussion, the authors often introduce experimental citations as explaining the modeling results, when really they should strive for the reverse, using the modeling to explain those experimental results. See L285: "the oscillation frequency increases with increased excitability, but decreases with increased inhibition, which may be due to that reducing GoC activity can stabilize high-frequency oscillations [45]."

A-II-10: Thanks for the comment. We have revised our text and discussion to incorporate the suggestions made by the Reviewer, and rephrased our arguments for the relationship between our modeling work and experimental data.

Q-II-11:While the methods are clearly written in regards to introducing modeling details, it would be helpful to summarize certain aspects of the model choices in the main text, particularly where model assumptions are likely critical to the conclusions.

For example, the presence and sources of heterogeneity are critical when discussing synchrony, but that detail is hidden deep in a methods table (I think heterogeneity is introduced only through threshold variability?)

A-II-11: Thanks for the suggestion. We revised our manuscript to include a summary of the model in the main text (see line 92).

Indeed, it is important to include noise and heterogeneity in the model. We do have many sources of heterogeneity in modeled neurons and synapses. Besides the threshold variability of neurons noted by the Reviewer, we also included the variability of weights in all types of synapses, as well as synaptic delays, and network layout of GoCs as gap junctions are depending on the distance between cells. The weight values we specified are the mean values, and we used a large variance around the means and sampled each synapse from a Gaussian distribution. We have now added more details about this point in the Methods Section.

Q-II-12:The authors should address whether they plan to release source code for their simulations for transparency and reproducibility. Building the simulations using one of the many widely used neural simulation packages would be additionally appreciated, as it simplifies reuse. If there were strong reasons not to do so, they might be worth mentioning.

A-II-12: We are always supporting Open Science. The code will be released and deposited as part of the publication. We will follow the standard guideline of PLoS Comput Biol, and list the source of our code at the dedicated place of PLos:

Data Availability: The code used to generate the results in this paper is available on https://github.com/jiankliu/GC-CoC-Network

Q-II-13: Numerous minor errors with word usage and sentence structure interfered with my reading of the manuscript, such that it's possible misunderstanding contributing to some of the issues raised above. I would advise the authors to get editing help from someone with full professional proficiency in English. A few examples in the author summary: "In the network" without introducing which network; "Feedforward inhibition is showed as"; "potentially for its paradigm- shifted role…".

A-II-13: Thanks a lot. We corrected these and other typos and grammar mistakes. We also had someone helped with proofreading to make the manuscript more readable.

Reviewer #3:

In the manuscript, the authors developed a new mathematical model to investigate

the putative functions of intercellular feedback and feedforward inhibitions in the oscillations of the cerebellar neural network. They first showed the firing patterns of a single granule cell (GC) can be altered when its interaction with a mossy fiber (MF) and a Golgi cell (GoC) follows three different inhibitory connections (FFI, FBI or FFI+FBI). Using this as a base, they simulated the oscillatory behavior of cerebellar neural network with inter-connected MFs, GCs and GoCs. Their results showed the importance of negative feedback (FBI) in generating network oscilations and the oscillatory frequency can be modulated by excitation and inhibition strength. Furthermore, they investigated how short-term plasticity and gap junction affect the network oscillations and how the three different inhibitory connections affect cross-frequency coupling of the cerebellar neural network. They concluded that different forms of feedback inhibition on GC play dominant roles in inducing network oscillation and in controlling cerebellar neural network's functions including cross-frequency coupling. This study postulates a new mathematical model that allows the investigation of cellular mechanisms underlying oscillatory behavior of the cerebellar neural network and can be a good reference for people in the field. However, I have several concerns regarding the design of their model and the interpretation of their simulation results. Please see the following for the major and minor points we raised for their work.

Major points:

Q-III-1: A previous experimental study (Holtzman et al., 2011) showed the existence of the inhibitory regulation from GCs to GoCs, whose function remains unknown. The current model can serve as an ideal way to evaluate the potential function of such an inhibitory regulation. I strongly suggest the autors to consider the inclusion of inhibitory regulation from GCs to GoCs and compare its effects with other feedback and feedforward inhibitions to make their work more complet and impactful.

A-III-1: Thanks for pointing out this study to us. If we are correct, the paper referred to by the Reviewer is:
Multiple extra-synaptic spillover mechanisms regulate prolonged activity in cerebellar Golgi cell–granule cell loops. (Holtzman et al., 2011)

We have gone through this study, where they proposed that there are multiple extra-synaptic spillover mechanisms from GCs to GoCs, including one type of mGluR2-mediated inhibition. We have carefully read the details in the paper and tried to found more details by checking other similar experimental studies following this study. We found that it is not easy to directly model this type of inhibition as it is mediated by mGluR2 synapse via the spillover across synapses, which cannot be simply modeled by a kinetic model of synaptic transmission. The above study indeed contributes to our understanding of the diversity of synaptic mechanisms in the granular layer and multiple time-scale dynamics discussed in the reviewing comment *Q/A-II-7*. The experiments of this study were conducted on the recording sites located in lobules

Crus II, and it was not clear if it exists in other lobules of the cerebellum, as it could be lobule-specific for certain types of synaptic behaviors. Thus, we take into account this study and discuss it with other relevant studies. Now we added a new part in the Discussion Section (see line 372 subsection – ‘Limitations’).

Q-III-2:For the construction of intercellular network, they adopted a published network topology. Since it is a fundamental and important feature of cerebellar neural network that can be hardly measured experimentally, it would be important to examine the effects of different cellular network topology and check how robust their results, e.g. cross-frequency coupling, will be.

A-III-2: Thanks for the comment. Here we used a 2D network topology in the model, since we need to include gap junctions, which require a distance measure between cells. Otherwise, ours is a random network topology when gap junctions are not considered. As noted by the Reviewer, measuring these properties is difficult experimentally. Thus, we adapted the network topology direction from a previous study, which has been constrained by experimental data. Indeed, it is important to take into account different network topologies, particularly when there are different localizations of cells in the cerebellum. For example, it may be useful to consider a modular network topology, where each subnetwork is a module, as we have discussed in *Q-II-7*. There are different localized lobules with different organizations of cells and network topologies. Extensive future work beyond the current work is needed to explore this point in detail. Here in the revision, we have conducted more analysis to confirm the robustness of our results (see Fig. S5, Fig. S8, and Fig.S11.)

Q-III-3: In Figures 6 and 7, the coupling patterns are scattered with some low coupling regions within high coupling regions (for example, dark blue bands within the white/yellow/red regions in the Fig.6B). What can be the cause of this discontinuity in the couplings?

A-III-3: Thanks for pointing it out. We have checked our analysis and found that this is due to that we only used the data of 1 second for these analyses and plotting. Now we have conducted longer simulations with 10 seconds of data, with which we replotted these figures (see Fig. 5 and Fig. 6, as well as Fig. S11).

Q-III-4:In Figure 5A, there is a pulsatile pattern of GCs with only FFI. Is the pulsatile pattern oscillation? It is likely that it is caused by the synchronization of neuronal activities by the gap junction that leads to seemly oscillatory patterns but not oscillation by its nature. Could the authors clarify this point by rigious statistical tests and modify their interpretation?

A-III-4: The reviewer is right that the case of FFI shown in Fig.5A has a weaker oscillation for GCs, and it is caused by the synchronization of GoCs through gap junctions. As in the FFI network, gap junctions between GoCs induce network

oscillation of GoCs themselves, and the GoC synchronization delivers a chunk of inhibition to GCs to form the pulsatile pattern of oscillation with low frequency. As noted by the reviewer, oscillation is not strong and not tight in the case of FFI. As shown in the plot of power-frequency, the oscillation has a low frequency around 10 Hz. We have conducted the additional analysis with different levels of the strength of gap junctions (see Fig. S8). We revised our text and modified our text about this point (see line 199).

Minor points:
Q-III-5:It would be helpful to explain how short-term plasticity is modeled in their text.
A-III-5-6-7: We have revised the text to explain it clear (see line 168).

Q-III-6:Line 74 and 83, "though" should be "through".
Q-III-7:The second line of the legend of Fig.7, "low filer" should be "low filter".
Thanks for correcting our typos. We have carefully checked typos and corrected them.
