# Supplementary material for: Regulating synchronous oscillations of cerebellar granule cells by different types of inhibition
Source: PLoS Comput Biol. 2021 Jun 28;17(6):e1009163. doi: 10.1371/journal.pcbi.1009163 (PMC8270418; doi:10.1371/journal.pcbi.1009163)
Supplement: Attachment — Submitted filename: PCB_rebuttel.pdf [file pcbi.1009163.s015.pdf]

PLOS Computational Biology (PCOMPBIOL-D-21-00018-R1)

Dear Editor and Reviewers:

First of all, we would like to express our gratitude to you for carefully reading our manuscript and giving us constructive suggestions. We have thoroughly considered your comments, and revised our manuscript in light of your remarks and suggestions. Thank you all for your comments during the revision of our manuscript.

The following are point-to-point responses to the reviewers' comments. The reply is arranged in Q's and A's. We first quote your comments, and then give our answers after each of your questions.

**Replies to the Comments from Editors:**

*Given the widely different comments received for the original submission, it has been decided to include one more reviewer.*
*For this revised version, reviewers 2 and 4 still raise important issues on the way in which the authors present and discuss their model and findings.*
*In this last round of review, the authors should adequately address these issues.*

We have carefully revised our manuscript in light of the comments provided by the reviewer 2 and 4. We revised our manuscript significantly to improve the clarity of the presentation and discussion of our model and findings.
We provided a version of the revised manuscript with highlights denoting where the text has been revised (in red) for easy reviewing. Please find the marked PDF file uploaded as '**Revised Article with Changes Highlighted.pdf**'. All the responses below indicated by line numbers refer to the marked PDF file rather than the clean copy PDF file.

**Reviewer #2:**

*Q-II-1:* (Relating to A-I-1, A-II-2, and overall novelty) – The revisions have helped clarify the distinction of the modeling methods from previous models, but still don't provide clear arguments that the new features of the network shown here (interaction between GoC and GC for stronger synchronized oscillations despite STP) are either a more realistic description of experimental observations or essential to a proposed function. What biological insight does this added complexity gain us?

A-II-1: Both aspects mentioned by the Reviewer are intertwined. The novelty is to provide a unified description of the contribution of GoC inhibition to GC oscillation. Our work certainly provides a more realistic description of experimental observations, e.g., how the oscillation of GCs is shaped by different effects of inhibition, feedforward inhibition, feedback inhibition, short-term plasticity, and gap junction, from GoCs. Such detailed examination of the granular network of GCs and GoCs has not been done. With these biological details included, our model is able to provide a testbed for the functional role of the granular network dynamics of the cerebellum, which is how the oscillation of GCs could contribute to more general phenomena of neural oscillations existing in not only Purkinje cells, but other interacted brain areas through cortico-cerebellar communication. The role of GC oscillation could be a basis for the communication of the cerebellum and the cortex, in line with the recent view of the novel roles of the cerebellum [De Zeeuw, Lisberger, Raymond. Nature Neuroscience 24 (2), 160-167]. Given the source of GC oscillation relies on GoC inhibition, the biological insight provided by our model is to test the different roles of GoC inhibition in neural oscillations by considering a wide range of experimental observations in the cerebellum and cortex. We have revised the text to make this point clear (see line 357).

*Q-II-2:* (A-II-1) – Although the additions are appreciated, they simply add complexity when the goal here should be to reduce it by teasing apart mechanisms, which I don't see progress on. Indeed, in (A-II-4) the authors seem to agree that a simple explanation is possible for the e/I effects on oscillation frequency. To me, that should be a reason to simplify the manuscript by passing over such results with a quick explanation and focus on the more novel results. Another place where I imagine the results could be considerably simplified with more careful analysis is in aligning the results on cross-frequency coupling with the results on oscillation frequency and strength when the same parameters are varied – do the coupling results directly follow the underlying oscillation in an intuitive way?

A-II-2: There seems to a misunderstanding from the Reviewer about our work. Our work is to examine diverse synaptic mechanisms of synchronous oscillations of GCs and GoCs. It is not about the simple E/I effects, rather, it is about synaptic mechanisms provide by GoCs. In terms of network oscillations, there is much theoretical work

examining various simplified versions of neural networks by ignoring biological details. Generic networks with E and I neurons have been examined in detail (see the work by Brunel [Journal of computational neuroscience 8 (3), 183-208 (2020)] and other works in the line). There are some theoretical works utilizing gap junctions for network synchronization, for which we added several more references [Ref. 1&2 below] in the revised text. By using much reduced and simplified settings, theoretical works could consider the network dynamics of oscillation with certain mechanisms, even the mechanisms themselves considered are much reduced and not comparable to those in biology. Similar is true for cross-frequency coupling. As our work here is not to reduce a network system to a single component for the mechanisms, and our aim is to provide many biological details for neurons and synapses and generate a testable model for later experiments. We do not see there is a way to simplify our manuscript by ignoring many desired biological details while keeping the diversity of synaptic mechanisms, e.g., there is no such a simple dynamical system with reduced coupled-oscillators mentioned by the Reviewer that can also take into account biological details we want to study here. The Reviewer may have a solution for this theoretical consideration, and we look forward to seeing it and happy to collaborate if possible. Thus, any theoretical simplification of our manuscript is out of the scope of our current work.

1. Chartrand, Goldman, Lewis. Synchronization of Electrically Coupled Resonate-and-Fire Neurons. SIAM J. Appl. Dyn. Syst., 18(3), 1643–1693. (2019)
2. Lewis, Rinzel. Dynamics of Spiking Neurons Connected by Both Inhibitory and Electrical Coupling. Journal of Computational Neuroscience volume 14, pages283–309 (2003)

*Q-II-3:* (Fig. S5, new issue) – The authors again add quite a bit of new complexity to show the robustness of the demonstrated suppression of oscillations for both depression and facilitation, but should more clearly explain this counterintuitive result, or focus on a single set of experimentally supported STP parameters. It appears that the least suppression is observed for small U, but that is not explained. Also, how much is explained simply by varying the mean value of u, and what is u fixed at for the no-STP case?

A-II-3: The robustness of model results was an essential point and was required by editors and other reviewers in the last round of revision. Manipulating the STP parameters systematically is necessary, as there is no single set of experimentally supported STP parameters. We believe the Reviewer agrees with us that the complexity of neural systems cannot be described by one single set of parameters in any models. Such a systematic exploration and manipulation is a typical modeling strategy used by the community, see Ref.1 below for example.

[1] Grangeray-Vilmint, et al. Short-Term Plasticity Combines with Excitation–Inhibition Balance to Expand Cerebellar Purkinje Cell Dynamic Range. Journal of Neuroscience, 38(22): 5153-5167 (2018).

In terms of smaller or larger U in the STP dynamics, it has been typically observed when modeling of STP that smaller U showing facilitation and larger U showing depression (Ref 1 above as well). u is a dynamic variable rather than a parameter, and it is controlled by U in STP. That is why we only need to vary U and show that the results are robust to the change of U.

As explained in our manuscript. We followed the standard protocol to model STP:

synaptic strength = synaptic conductance G * u

where G is also called synaptic weight used in our work. For the no-STP case, u=U is a fixed value as we have described in our manuscript. The Reviewer pointed out that one can simply vary u (or U), however, as noted above, changing u (or U) for the no-STP case is exactly equivalent to varying G for the overall synaptic strength. In our original manuscript, we have already symmetrically varied G. That is why there is no need to vary u (or U) for considering the effect of synaptic strength for the no-STP case.

*Q-II-4: Minor points: (A-I-3) – There seems to have been some confusion here between reviewers and authors between STP and STDP. While STDP is certainly out of scope, that should probably be clearly stated in the manuscript to avoid confusion.*

A-II-4: Indeed, STP and STDP are quite different. STP has been generally observed in the synapses of various types of neurons in the cerebellum, while STDP is much less observed so far. However, a recent study shows the existence of STDP at the rat cerebellar mossy fiber–granule cell synapse [Sgritta, Locatelli, Soda, Prestori and D'Angelo. Journal of Neuroscience 2017, 37 (11) 2809-2823]. We added some text about this point at line 464, see below.

"Besides short-term plasticity, another form of frequency-dependent plasticity is spike-timing-dependent plasticity that was found recently at the MF-GC synapses with a preferred frequency of 6-10 Hz [84]. This additional factor may also contribute to the synchronous oscillation of the GC population. The exact functional implication of this new finding remains to be elucidated by more detailed experimental and modeling studies."

**Reviewer #4:**

*Q-IV-1: The network model presented here is solely based on a prior report from a different group (Dugue et al Neuron 2009). It was suggested to compare it to other models or systems. This has not been done and remains a critical point. Is this model robust enough that the conclusions remain true if based on other experimental data?*

A-IV-1: Sorry for the confusion. Our network model is not solely based on the prior report (Degue et al Neuron 2009 – D09). Our network model has a few more mechanisms included, and gap junction is only one of them. For gap junction between GoCs, we did use experimental data of D09. However, the work of D09 only modeled GoCs and did not model GCs. Our model here is different, as we model the network of both GoCs and GCs. When there is no gap junction in the network, our model of GCs and GoCs does not require the data of D09. As explained in the last revision, when there is no gap junction, our network is a random network and has no specific network topology. 2D network setup is only necessary for gap junction, and it is the typical modeling setup for other models and systems as well. Thus, compared to other models/systems, our network model is a model that unifies separate works of GCs and GoCs, as well as individual synaptic mechanisms, using experimental data and data-validated model components. We have revised our text in Methods to make it clear.

In our last round of revision, we have added more robustness results about gap junction (Supplemental Fig.S8). For gap junction, it is a relatively simple phenomenon in terms of modeling, and the gap junction strength is dependent on the distance between cells. The key parameter is the overall strength or gap junction conductance. By varying it to be stronger or weaker, our results are robust enough and the conclusions remain true. Thus, for other experimental data where gap junctions are different, our conclusions will remain true, and our model can be adapted to model those specific systems and experiments as well.

*Q-IV-2: The term 'oscillations' remain confusing (e.g., see former Q-II-9 comment). While the fact that these are meant always to be synchronous oscillations may be obvious for readers from a narrow field, it is important to make this difference and the paper's conclusions clear to a wider audience. A few sentences in the intro or results section should suffice.*

A-IV-2: Thanks for the suggestion. We have revised our manuscript title, abstract, summary, introduction, and other necessary parts, to repress the term as 'synchronous oscillation', to make this point clearer. We also included a few texts in the intro (line 58), see also below:
'In contrast to the oscillation at the level of single neurons, where intrinsic cellular

properties, such as ion channels, play a dominant role [13,14], synchronous oscillation at the network level involves synaptic interactions between neurons.’

*Q-IV-3: Q-II-11 remains a concern - a better explanation of the model and the assumptions clearly explained at the beginning of the results section is needed, especially for a wider audience. The details remain scant in the main text and the methods section will be too intimidating for a non-computational biologist. This will reduce the ability of many readers to interpret this model and limit the impact of the paper.*

A-IV-3: We have further revised this part to better summarize the model at the beginning of the results section. Note that according to the publication format of PLoS Computational Biology, we have moved the Methods section before the Results section. To avoid repeated descriptions for methods, we provide a concise summary at the beginning of the results section in the revised text (see line 186). Since the gap junction was only introduced and used later, we summarized it only when it was used in Sec C in the results section. We also revisited our Methods part to make it more readable for non-computational biologists.
