# Supplementary material for: Regulating synchronous oscillations of cerebellar granule cells by different types of inhibition
Source: PLoS Comput Biol. 2021 Jun 28;17(6):e1009163. doi: 10.1371/journal.pcbi.1009163 (PMC8270418; doi:10.1371/journal.pcbi.1009163)
Supplement: Attachment — Submitted filename: PLoS_CB_review_comment_SC.docx [file pcbi.1009163.s013.docx]

In the manuscript, the authors developed a new mathematical model to investigate the putative functions of intercellular feedback and feedforward inhibitions in the oscillations of the cerebellar neural network. They first showed the firing patterns of a single granule cell (GC) can be altered when its interaction with a mossy fiber (MF) and a Golgi cell (GoC) follows three different inhibitory connections (FFI, FBI or FFI+FBI). Using this as a base, they simulated the oscillatory behavior of cerebellar neural network with inter-connected MFs, GCs and GoCs. Their results showed the importance of negative feedback (FBI) in generating network oscilations and the oscillatory frequency can be modulated by excitation and inhibition strength. Furthermore, they investigated how short-term plasticity and gap junction affect the network oscillations and how the three different inhibitory connections affect cross-frequency coupling of the cerebellar neural network. They concluded that different forms of feedback inhibition on GC play dominant roles in inducing network oscillation and in controlling cerebellar neural network’s functions including cross-frequency coupling. This study postulates a new mathematical model that allows the investigation of cellular mechanisms underlying oscillatory behavior of the cerebellar neural network and can be a good reference for people in the field. However, I have several concerns regarding the design of their model and the interpretation of their simulation results. Please see the following for the major and minor points we raised for their work.

Major points:

1. A previous experimental study (Holtzman et al., 2011) showed the existence of the inhibitory regulation from GCs to GoCs, whose function remains unknown. The current model can serve as an ideal way to evaluate the potential function of such an inhibitory regulation. I strongly suggest the autors to consider the inclusion of inhibitory regulation from GCs to GoCs and compare its effects with other feedback and feedforward inhibitions to make their work more complet and impactful.
2. For the construction of intercellular network, they adopted a published network topology. Since it is a fundamental and important feature of cerebellar neural network that can be hardly measured experimentally, it would be important to examine the effects of different cellular network topology and check how robust their results, e.g. cross-frequency coupling, will be.
3. In Figures 6 and 7, the coupling patterns are scattered with some low coupling regions within high coupling regions (for example, dark blue bands within the white/yellow/red regions in the Fig.6B). What can be the cause of this discontinuity in the couplings?
4. In Figure 5A, there is a pulsatile pattern of GCs with only FFI. Is the pulsatile pattern oscillation? It is likely that it is caused by the synchronization of neuronal activities by the gap junction that leads to seemly oscillatory patterns but not oscillation by its nature. Could the authors clarify this point by rigious statistical tests and modify their interpretation?

Minor points:

1. It would be helpful to explain how short-term plasticity is modeled in their text.
2. Line 74 and 83, “though” should be “through”.
3. The second line of the legend of Fig.7, “low filer” should be “low filter”.
